# Unique Device Identification–Based Linkage of Hierarchically Accessible Data Domains in Prospective Surgical Hospital Data Ecosystems: User-Centered Design Approach

**DOI:** 10.2196/41614

**Published:** 2023-01-27

**Authors:** Karol Kozak, André Seidel, Nataliia Matvieieva, Constanze Neupetsch, Uwe Teicher, Gordon Lemme, Anas Ben Achour, Martin Barth, Steffen Ihlenfeldt, Welf-Guntram Drossel

**Affiliations:** 1 Center for Evidence-Based Healthcare Technische Universität Dresden Dresden Germany; 2 Fraunhofer Institute for Machine Tools and Forming Technology IWU Dresden Germany; 3 Professorship for Adaptronics and Lightweight Design in Production Technische Universität Chemnitz Chemnitz Germany; 4 Fraunhofer Institute for Ceramic Technologies and Systems IKTS Dresden Germany; 5 Chair of Machine Tools Development and Adaptive Controls Technische Universität Dresden Dresden Germany

**Keywords:** electronic health record, unique device identification, cyber-physical production systems, mHealth, data integration ecosystem, hierarchical data access, shell embedded role model

## Abstract

**Background:**

The electronic health record (EHR) targets systematized collection of patient-specific, electronically stored health data. The EHR is an evolving concept driven by ongoing developments and open or unclear legal issues concerning medical technologies, cross-domain data integration, and unclear access roles. Consequently, an interdisciplinary discourse based on representative pilot scenarios is required to connect previously unconnected domains.

**Objective:**

We address cross-domain data integration including access control using the specific example of a unique device identification (UDI)–expanded hip implant. In fact, the integration of technical focus data into the hospital information system (HIS) is considered based on surgically relevant information. Moreover, the acquisition of social focus data based on mobile health (mHealth) is addressed, covering data integration and networking with therapeutic intervention and acute diagnostics data.

**Methods:**

In addition to the additive manufacturing of a hip implant with the integration of a UDI, we built a database that combines database technology and a wrapper layer known from extract, transform, load systems and brings it into a SQL database, WEB application programming interface (API) layer (back end), interface layer (rest API), and front end. It also provides semantic integration through connection mechanisms between data elements.

**Results:**

A hip implant is approached by design, production, and verification while linking operation-relevant specifics like implant-bone fit by merging patient-specific image material (computed tomography, magnetic resonance imaging, or a biomodel) and the digital implant twin for well-founded selection pairing. This decision-facilitating linkage, which improves surgical planning, relates to patient-specific postoperative influencing factors during the healing phase. A unique product identification approach is presented, allowing a postoperative read-out with state-of-the-art hospital technology while enabling future access scenarios for patient and implant data. The latter was considered from the manufacturing perspective using the process manufacturing chain for a (patient-specific) implant to identify quality-relevant data for later access. In addition, sensor concepts were identified to use to monitor the patient-implant interaction during the healing phase using wearables, for example. A data aggregation and integration concept for heterogeneous data sources from the considered focus domains is also presented. Finally, a hierarchical data access concept is shown, protecting sensitive patient data from misuse using existing scenarios.

**Conclusions:**

Personalized medicine requires cross-domain linkage of data, which, in turn, require an appropriate data infrastructure and adequate hierarchical data access solutions in a shared and federated data space. The hip implant is used as an example for the usefulness of cross-domain data linkage since it bundles social, medical, and technical aspects of the implantation. It is necessary to open existing databases using interfaces for secure integration of data from end devices and to assure availability through suitable access models while guaranteeing long-term, independent data persistence. A suitable strategy requires the combination of technical solutions from the areas of identity and trust, federated data storage, cryptographic procedures, and software engineering as well as organizational changes.

## Introduction

Unique device identification (UDI) is a system used to identify devices within the health care supply chain based on a consistent, standardized, and unambiguous machine-readable identifier to keep track of the postmarketing performance of medical devices [1]. The performance of a hip implant, for example, cannot be evaluated without considering individual factors of the recipient [2] and the conditions of the therapeutic intervention [3]. Consequently, patient, medicine, and product have to be linked and monitored to enable a well-founded evaluation. Regardless of the still-missing legal framework conditions [4] and the existing ethical and political questions [5], digitalization of the health system is advancing [6], which is beneficial for a more holistic assessment. Either way, part of this development is the cross-domain linkage of data [7] that at least can be resolved on a patient-by-patient basis to prepare for intelligent data analysis. This requires going beyond analysis objectives to an appropriate data infrastructure, which enables different data domains to be linked while providing adequate hierarchical data access concepts. Consequently, this paper approached a framework for cross-domain cooperation and intelligent data analysis in a specific application scenario embedded in prospective digital hospital ecosystems. Linking unconnected domains inside of safe frameworks with clarified data sovereignty to enable a holistic approach to personalized treatment benefits health care, lowers risks of mistreatments, and functions as a catalyst for the optimization of medical products. This concept, based on a pilot scenario, can function as a basis to clarify unclear legal issues.

## Methods

### Framework Conditions for Digitization in Individualized Medicine

#### Intelligent Data Analysis Framework

**Figure 1:**
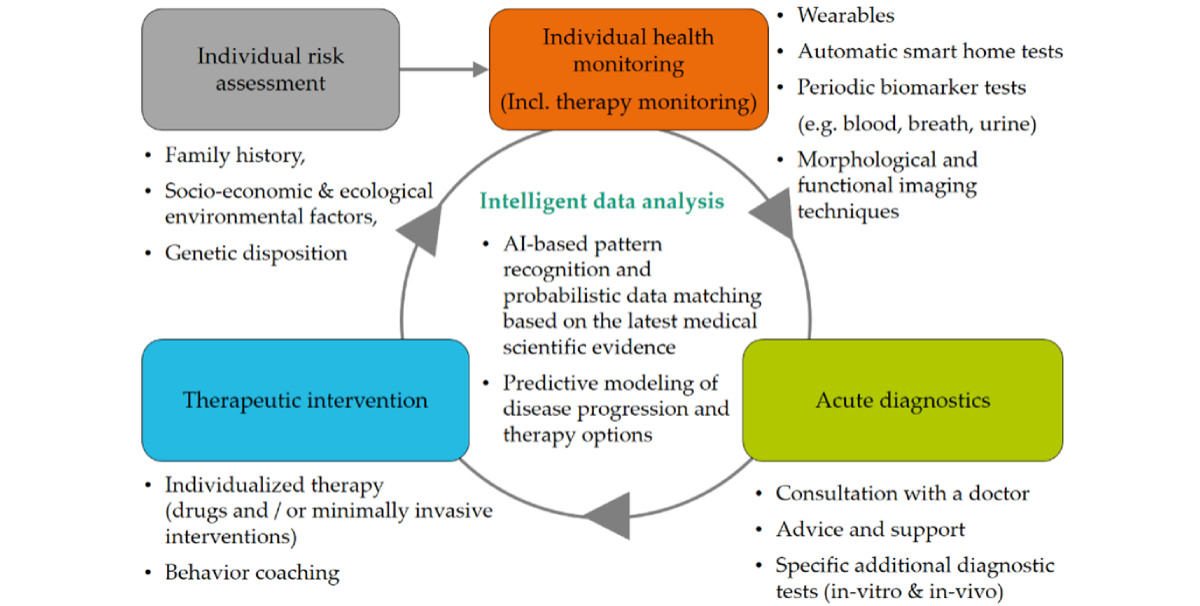
Information cycle of individualized medicine [9]. AI: artificial intelligence.

An EHR is the systematized collection of electronically stored patient and population health data in a digital format. These records can be shared across different health care settings [8]. Records are provided through network-connected, enterprise-wide information systems or other information networks and exchanges. EHRs can include a range of data such as individual risk assessments, health monitoring, acute diagnostics, and therapeutic interventions while enabling comprehensive, intelligent data analysis, which, according to Hahn et al [9], is considered the information cycle of personalized medicine (Figure 1).

These data are stored as a patient digital twin in a centralized hospital information system (HIS) in the form of text, images (DICOM and other types), and scans. EHRs enable patients and hospitals to manage their health information in public (eg, hospital) and private environments as a personal health record (PHR). The information contained in EHRs is highly sensitive. Unintended exposure of these data threatens an intimate part of a patient's private sphere and may lead to undesirable consequences. The EHR is a communication tool that supports clinical decision-making, coordination of services (illness type, care type), evaluation of the quality and efficacy of care, research, legal protection, education, and accreditation, and regulatory processes. It is the business record of the health care system, documented in the normal course of its activities. Patients routinely review EHRs and keep PHR in their own digital archive or in patient portals (eg, at health insurance companies such as “TK-Safe,” “Vivy,” or “AOK-Gesundheitsnetzwerk” in Germany), given the patient is the owner of the EHR and PHR. The physician, practice, or organization is the owner of the physical medical record because it is its business record and property, and the patient owns the information in the medical record. Although the record belongs to the care facility or doctor, it is truly also the patient's information. EHRs should be released to other stakeholders only with the patient's permission or as allowed by law or via studies: public registry and ministries. PHRs are already on the market. The purpose of a PHR is to maintain good health and target outcomes; this could include daily vital signs (eg, blood pressure, heart rate), number of walking steps, amount of exercise, and calorie intake. In addition to these, information for medical use might be considered, such as blood type, allergies, pre-existing diseases, medicines the user is taking, emergency contact information, and information about the user's medical institution.

#### Synergy Potential in Information Linkage Using the Example of a Hip Implant

**Figure 2:**
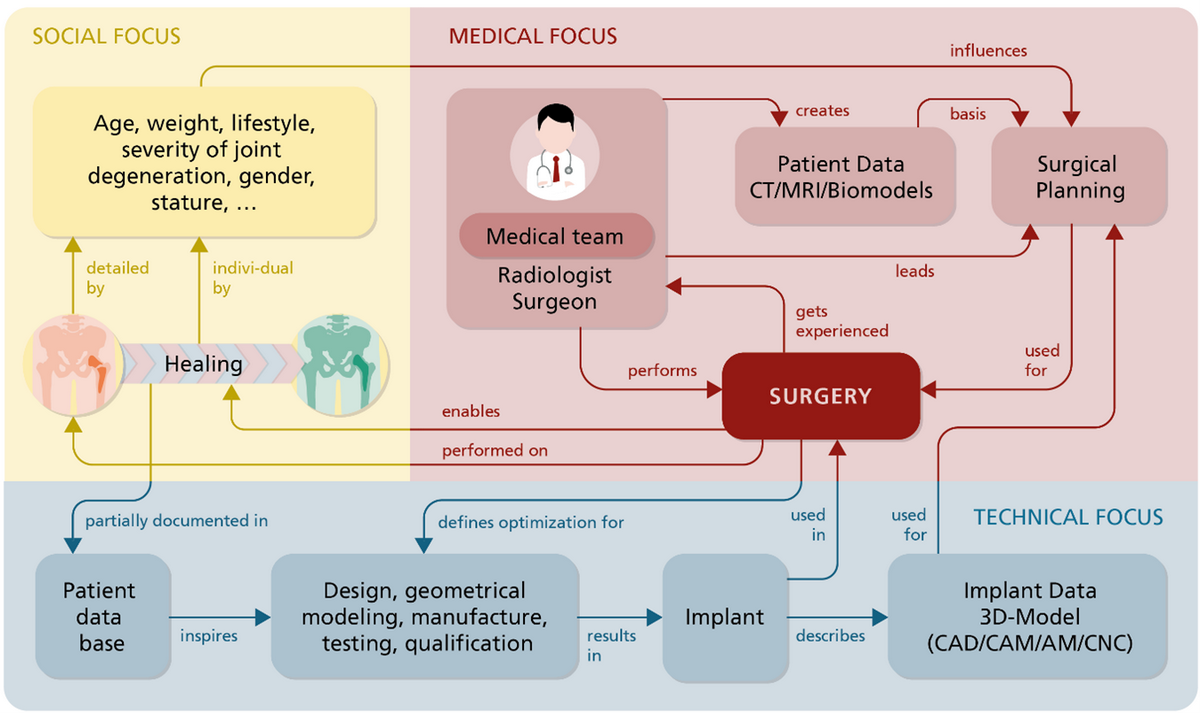
Incident-based networking of social aspects, medical factors, and technical aspects. AM: additive manufacturing; CAD: computer-aided design; CAM: computer-aided manufacturing; CNC: computer numerical control; CT: computed tomography; MRI: magnetic resonance imaging.

Neugebauer [10] stated that the digital transformation is promoted by the interaction of technologies that were previously perceived as independent of each other. Hahn et al [9] noted, in this context, that networking of the individual sectors and the structured use of integrated information are still pending. Moreover, Hahn et al [9] concluded that sustainable success can only be expected if the interlinking of technological and biomedical research on the one hand and clinical implementation and product development on the other hand are permanently guaranteed. Either way, this paper approached data-driven interdisciplinary research from an application-oriented perspective in an incident-based scenario (Figure 2). This example illustrates selected dependencies between social parameters, medical factors and technical aspects important for surgery, and healing, which are currently not linked sufficiently. In fact, it is a common practice to laboriously obtain this information on a case-by-case basis, which requires appropriate lead time before the operation; this is a major disadvantage in the case of emergency medical treatment. Consequently, this paper addressed this shortcoming and developed a possible solution scenario showing how this information can be linked at the EHR level. Moreover, we’ve shown how this information can be made accessible based on implant-inherent features while introducing a role model for access regulation and data protection.

### Data-Driven Networking of Information

#### Regulatory Demands

**Figure 3:**
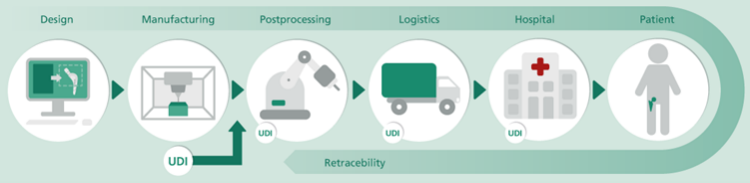
Retraceability of a medical device with a unique device identification (UDI).

In the medical technology industry, quality assurance is a particular focus due to the stringent regulations. With a changeover period, the European Medical Device Regulation (MDR) EU 2017/745 came into effect in May 2021 and replaced the European directives on medical devices. The regulation obligates manufacturers to mark medical devices that are marketed in the European Union with unique codes. The main objective for the introduction of these codes is to increase patient safety. Unique product identification prevents confusion of medical devices and makes counterfeiting more difficult. The markings are implemented by the UDI system. The UDI enables tracking of medical devices, for example, from the last step of postprocessing in manufacturing where the marking is applied to the component (eg, by laser engraving). Nevertheless, the medical products can only be tracked from this point through the logistics process to the hospital. Figure 3 illustrates selected phases of a medical device life cycle covering design, manufacturing, postprocessing, logistics, and the union with the patient. Unfortunately, doubtless identification of an implant after implantation is impossible with the UDI system, which largely excludes proof of quality and originality after implantation. Across industries, product piracy is a major problem, and the estimated economic damage has been increasing over the years [11]. In addition to the generally valid comments on product piracy and its consequences, other aspects have to be considered in the field of medical technology. After the publication of the “Implant Files” in 2018 by a group of investigative journalists, the problem of defective medical devices became a sociopolitical issue [12].

#### Component-Inherent Identifier–Based Data Access

**Figure 4:**
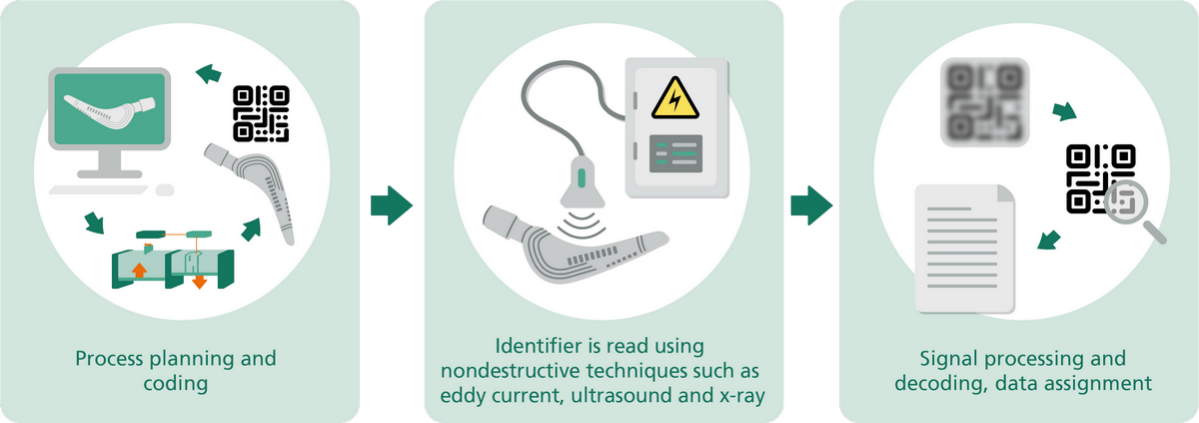
Principle of inherent markings in an additively manufactured implant with part identification and tracking.

Inherent markings of implants could be a solution to prevent counterfeiting while satisfying the regulatory requirements for a machine-readable marking in the form of a barcode or data matrix. In addition, traceability can be expanded back to the design process when the inherent markings are integrated. Moreover, this simultaneously enables inclusion of the manufacturing process into the traceability chain as well (see Figure 3). Either way, the greatest potential for innovation is seen in the ability to clearly identify the implant after surgery, preferably via noninvasive technologies already available in the hospital or technologies that can be provided without great technological effort and financial investment. Either way, the inherent feature could act as a key to access distributed information (see Figure 2) after signal processing and appropriate decoding (Figure 4).

Matvieieva et al [13] performed an identifier read-out applying eddy current (EC), ultrasound (US), and micro-computed tomography (CT; also in x-ray mode) as nondestructive methods. The EC, US, and x-ray techniques allow the receipt of part of the inherent data encoded in 1D and 2D codes. The feasibility of the methods was shown for 1D Pharmacode, UDI 1D Barcode (ISO 128), and UDI 2D DataMatrix for titanium, titanium alloy, and stainless steel. Due to the physical restrictions of the chosen nondestructive methods (eg, penetration depth, magnetic and acoustic wave transmission, propagation, and density), the obtained identifiers’ signals have to be processed. In fact, after the identifier´s read-out, the obtained signals are processed by morphological and mathematical operations; once decoded, they enable further linkage with data or information stored in a database (Figure 4).

#### Technical Focus Data

**Figure 5:**
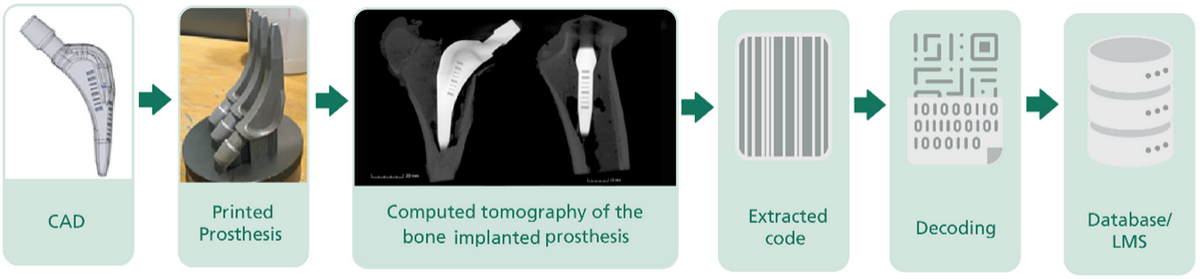
Example of the component (part) identification using a Pharmacode integrated into the hip prosthesis and data extraction from the identifier in the implanted state by computed tomography. CAD: computer-aided design; LMS: laboratory management system.

**Figure 6:**
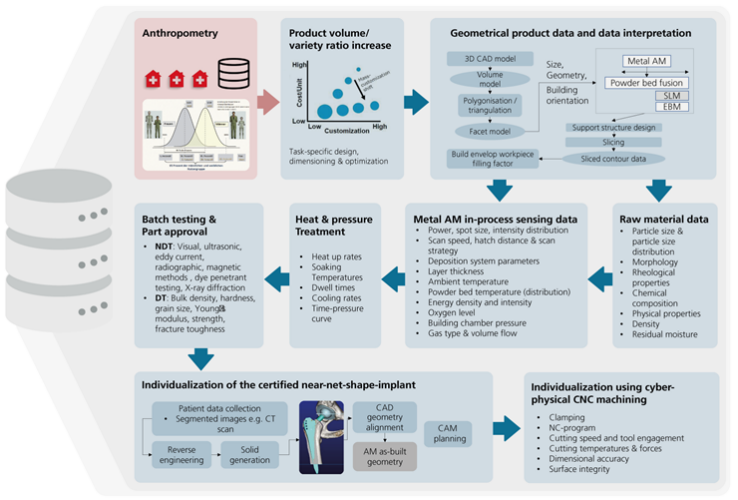
Schematic illustration of the process chain for additive manufacturing and subtractive individualization of hip implants with a selection of quality-determining parameters that are monitored and archived in process databases as part of quality assurance. AM: additive manufacturing; CAD: computer-aided design; CAM: computer-aided manufacturing; CNC: computer numerical control; CT: computed tomography; DT: destructive testing; EBM: electron beam melting; NC: numerical control; NDT: nondestructive testing; SLM: selective laser melting.

The starting point is the construction of a 3D model of a hip implant (Figure 5) based on anthropometric data or even patient-specific information in which the coding is also integrated. Considering typical hip implant sizes, geometric complexities, and quantities, 3D printing (additive manufacturing [AM]) is increasingly developing as a competitive manufacturing method [14]. Suitable AM procedures are primarily selective laser melting (SLM) [15,16] or electron beam melting [17,18]. In addition, layer-wise build-up and achievable resolution are very beneficial for the integration of inherent features during manufacturing (see Figure 3). Using these manufacturing processes, the 3D data model needs to be converted into a facet model first (mesh) [19]. Then, the parts are positioned in the build chamber, supported, and sliced using standard AM software [20]. Based on material selection criteria such as biocompatibility, the Young modulus, strength, and fatigue strength, a (certified) raw material is selected [21-23] that is particularly tailored to the AM process requirements [24]. Material selection criteria like particle size, particle size distribution, and morphology or chemical properties are continuously checked and monitored [25,26]. The SLM process data, for example, have to be qualified for applications like implants and are subdivided into predefined parameters (considered static) and parameters to be controlled by in-process sensing (considered dynamic) [27]. Either way, essential parameters are monitored and archived [28-30]. The same applies to processing data from postheat or pressure treatment [31] as well as from destructive and nondestructive material testing [32]. Moreover, mechanical postmachining, performed to adapt the implant to the recipient needs, generates data [33]. An example of this is the description of the geometric interface to the patient (bone-implant interface) including parameters such as surface roughness. The result, however, is an extensive description of the implant and the implant creation process as well as additional information such as corresponding implant tools (Figure 6). Obviously, some of this information is of interest to surgeons (medical focus), whereas information about the implant recipient and the use of the implant (social focus) are of interest to the manufacturer (Figure 2). This means, regardless of the individual legal framework, the possibility of controlled linkage of information is seen as desirable.

#### Social Focus Data

**Figure 7:**
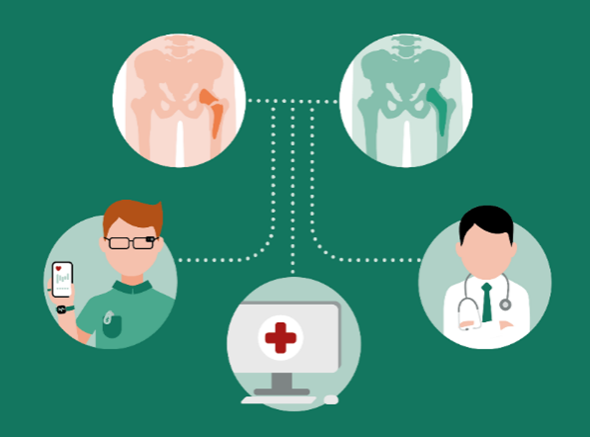
Illustration of close monitoring of healing using mobile health (mHealth) applications.

The social background of an implant-receiving patient (Figure 2) includes individual characteristics and conditions such as age, gender, lifestyle, and constitution type, which is of crucial importance for the formation of diseases, their duration, and treatment. The continuous monitoring or even documentation of this background can be ensured by a variety of technologies from the field of mobile health (mHealth), which allow broad mapping of dynamic data sets such as lifestyle and physical activities [34]. mHealth is an aspect of eHealth, although there is no universal definition of mHealth [1]. However, there is consensus that mHealth can be understood as medical and public health practice supported by mobile devices, such as mobile phones, patient monitoring devices, personal digital assistants, and other wireless devices [35]. This means that mHealth can be understood as “the use of mobile communications for health information and services” as patient-individual behavior without direct involvement of the health service provider [36]. Here, mHealth is seen as the technical prerequisites to monitor and document the social focus data during healing (Figure 7) or even beyond (see Figure 2).

In addition to the fact that mobile communication and audiovisual interaction are the decisive enablers for mHealth, there is also the aspect of powerful sensor-based hardware and high flexibility in software development for smartphones and, to a limited extent, wearables [37]. In contrast to smartphones, wearables can be designed very specifically and can therefore be used for special applications (eg, sleep monitoring) [38]. Widespread sensor systems for smartphones include [39-41] light sensor technology (ambient light, camera system in combination with lighting), proximity sensors, acceleration sensor technology, rotation sensor technology (gyroscope), electromagnetic sensor technology, digital compass (magnetometer), acoustic sensor technology via microphone, and sensor technology for location tracking (GPS).

In contrast to smartphones, wearables are more specific for a particular application and therefore have higher specificity for the integrated sensors, so that headbands can, for example, derive targeted electroencephalography [42]. This means that targeted data collection is possible with the help of sensor technology, internal processing by specific software (eg, apps), visualization based on these data, and mostly wireless communication to third parties in the form of a uniform data image. For example, the following parameters can be acquired by means of wearables and mobile devices: heart rate and pulse oxymetry readings with photoplethysmography [43] and systems to monitor activity and sleep [11].

Hence, mHealth is seen as an enabler that contributes to rehabilitation by providing valuable data about the rehabilitation measures and patient-specific activities (Figure 6) that can be stored in the EHR. In addition, realistic load scenarios can be determined that could contribute to the further optimization of hip implants (Figure 2)—to promote healing [44] while targeting shorter hospital stays and lower treatment costs, for example [45]. In addition, mHealth can help achieve a consistent database (Figure 2) to evaluate the optimal intensity, frequency, and effects of rehabilitation from a wide variety of patients over a longer period of time as these data are currently not available or insufficient [46]. Either way, linking social focus data and production data could enable significant improvements with regard to determining the actual wearing of the implant, for example, based on characteristics such as posture, weight, and movement profiles, which are summarized here as social focus data (Figure 2).

#### Medical Focus Data

Both routine diagnostics and revision surgery require information about implants that were placed decades ago. Specific identification using only medical imaging is currently not possible and requires access to the documentation by the initial treating physician or hospital (Figure 2). For elective as well as acute medical interventions, this documentation is not available or only available with enormous effort. With inherent markings in the implant, it is feasible to obtain information relevant for revision surgery or routine diagnostics even after the insertion of an implant into the human body [47] (Figure 4). To assure valid and reliable surgical planning, medical data from past treatments, especially the surgical processes, follow-up examinations, and rehabilitation measures, are needed. Insufficient information about the implant and medical focus data (Figure 2) could lead to complications during revision surgery; therefore, considerable efforts are being made to obtain this information, which significantly extends the preoperative time in the hospital and results in additional costs. In fact, an example of the necessary information about the particular implant concerns the appropriate revision instruments and the existing implant components [48-50]. Moreover, the research effort prior to the operation is continuously increasing, linked to the increasing number of operations and growing variety of implant types, sizes, variants, and material combinations. In addition to knowledge on the implant system used, information on the initial implantation process, which cannot be seen in medical images (CT, x-ray), is highly relevant for a gentle and successful revision of a hip implant. In fact, insufficient information clearly increases the risk of complications for the patient. Here, it shall be emphasized that the use of unsuitable revision instruments causes the risk of an enlargement of the wound surface resulting from an invasive procedure. This, in turn, increases the risk of infection and bleeding or even periprosthetic fracture. In addition, the inevitable prolongation of the surgery time can lead to a higher anesthetic risk, increased risk of thrombosis, and unstable cardiovascular function. Consequently, there is the obligation to report “incidents” in connection with medical devices to the German Federal Institute for Drugs and Medical Devices (BfArM), which includes an indication of the cause. Hence, unique inherent identification (Figure 4) and (partial) networking of information (Figure 2) in a transparent and retraceable database seem promising to assure a higher quality of health care [11], if unauthorized access is avoided.

## Results

### Database and Data Access

#### Data Integration From Heterogeneous Domains

Data integration is a crucial issue in the environment of heterogeneous patient-production data sources (Figure 2). First, there are heterogeneous data types and formats located in different databases, which implies that solving data integration challenges is a prerequisite for gaining useful information and knowledge based on appropriate analytical methods. Although concepts for databases to process heterogeneous data sets exist [51], the Laboratory Management System 4.0 (LMS 4.0) was developed specifically for the purpose of taking into account nonmedical stakeholders.

**Figure 8:**
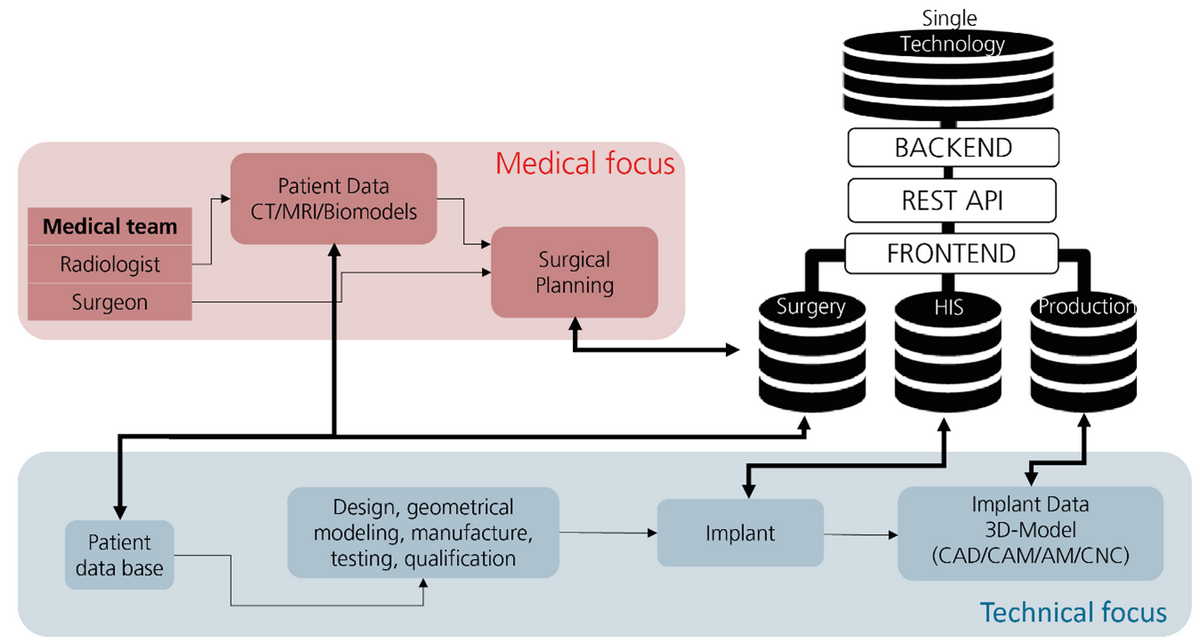
Partial networking of distributed data and information from different domains. AM: additive manufacturing; API: application programming interface; CAD: computer-aided design; CAM: computer-aided manufacturing; CNC; computer numerical control; CT: computed tomography; HIS: hospital information system; MRI: magnetic resonance imaging.

Exemplary implementation and application of this database structure were successfully demonstrated for the case of operations on the lip-jaw-palate region [52]. LMS 4.0 enables requesting data from different locations (eg, surgery, the HIS, or an implant producer) as a routine using web user interfaces. Using LMS 4.0, the surgeon collects magnetic resonance imaging results, for example, from HIS, checks patient data stored there, and has access to the integrated technical focus data (Figure 8) while planning the operational approach.

Based on integrated patient and production data, which can be expanded to an implant database that covers several manufacturers, surgical planning is simplified in order to determine which bone implant is the most suitable in the particular case, for example. Moreover, LMS 4.0 generates a dashboard and report that help the operating staff prepare for the surgery with the selected implants through the automatic output of the associated tools and instruments. Moreover, the surgeon can use this report to prove his or her preparations for the procedure and comprehensively explain the operation to the patient. LMS 4.0 presents an architecture that implements data integration in the hospital from the production, surgery preparation, and patient data. LMS 4.0 integrates databases without any changes to the individual databases (SQL database, software back end, application programming interfaces [APIs], front end) nor any need to maintain another database. The solution combines database technology and a wrapper layer known from extract, transform, load (ETL) systems and brings it to the SQL database, WEB API (back end) layer, interface layer (REST API), and front end. It also provides semantic integration through a connection mechanism between data elements. The solution allows for integration of patient, surgery, and production data in one technological framework: data management platform and implementation of analytical methods in one end user environment. The patient data (see Figure 2) are transferred, securely, to a HIS. Medical data storage in LMS 4.0 offers a highly scalable clinic web storage service that uses cumulative digital objects (eg. patient, surgery, implant) rather than blocks or files. Object storage typically stores data, along with metadata that identify and describe the content. For metadata management and automated quality control and data fusion (ETL processes), a data consistency model (LMS I4.0 metamodel) is used to enable eventual consistency for updates or deletes to existing objects.

#### Role Concept for Secure Data Access

**Figure 9:**
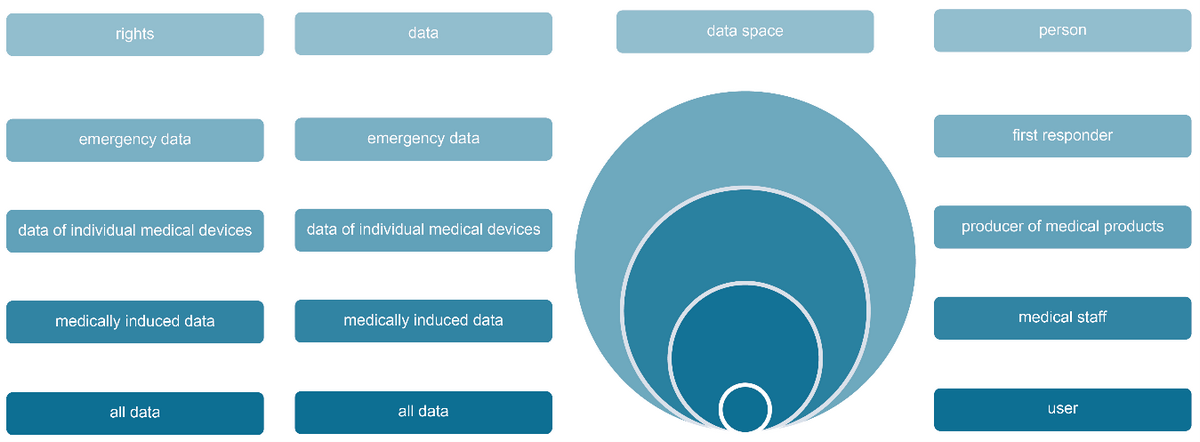
Principle of hierarchical data access based on a shell embedded role model.

On the basis of the information domains and the links and interfaces shown in Figure 2, it becomes obvious that the database structure and the information technology (IT) system design in the back end (see Figure 8) have to accommodate different user roles to protect secure data access to sensitive patient data. Consequently, a role model was developed that takes into account both different users or user groups (eg, patients, medical staff, manufacturers of medical products, and first aid providers) as well as special situations (eg, emergency access). Deviating from static access models (role-based access control) as well as traditional shell models in this case, pure login information was linked with additional contextual information (attributed-based access control) in order to allow hierarchical access control. Either way, the principle is shown in Figure 9, indicating the 4 basic roles embedded in the defined shell model.

Nevertheless, the person category is subdivided into 4 roles: “first responder,” “producer of medical products,” “medical staff,” and “user.” Users can view all data via a terminal device after registration, for example, with a digital health card equipped with a radio-frequency identification transponder (radio-frequency identification), and allowed to only make entries in a dedicated area of the social focus data section (Figure 2). The write permissions include data integration from fitness watches, training performance in rehab or sports facilities, and self-collected nutritional data, while interfaces to cell phone apps, for example, are available to substitute manual input. On the other hand, the medical staff can read medically relevant data and has the right to make entries in the medical focus data (Figure 2), which shows who made the entry. This corresponds to an entry in the EHR stored in the HIS (Figure 7). Producers of medical products only have access to the medical device data area, summarized as production (Figure 7). Technical focus data (Figure 2; eg, appropriate revision instruments as described in the Medical Focus Data section) are stored here, while the patient and implant are linked in the medical focus database (Figures 2 and 7). This information is requested from the manufacturer via a modified procurement process, which ensures that the agreed data are available before the invoice for the implant is paid. Consequently, all relevant product information is stored, enabling the simplification of follow-up treatments, support for minimally invasive interventions, and excluding of medical interaction. The information transfer to the manufacturer (see the Technical Focus Data section), on the other hand, can be enabled using a data integration center with a data use and access committee for research inquiries, as is currently being developed by Prokosch et al [53]. The last role in the person category is the first responder (Figure 8), which is introduced to explain the dynamic access approach. The first responder occurs in case of an accident or emergency when lifesaving measures, for example, are necessary. For example, access is granted for a certain period if several predefined factors that were detected using a fitness watch or other smart device take effect at the same time (eg, oxygen saturation in the blood, blood pressure, or other health-endangering characteristics). However, these attributes are securely transferred to the LAB 4.0 database management system (see the Data Integration From Heterogeneous Domains section) to obtain the necessary information depending on the authorization or to allow the addition of data. For this purpose, a standardized, well-defined interface is used to realize the data exchange and integrate smart devices for pure information retrieval as well as to develop software extensions that can be used to store the data in the database while complying with access restrictions. This enables the creation of a digital ecosystem for different participants to provide patients with optimal and, above all, digital, end-to-end health care while providing adaptive access regulations meeting authenticity requirements and assuring authenticity and appropriate access tracking. A method of secure patient-centered management of EHR data, though it can also be further processed in a deidentified format for statistical purposes, has been demonstrated with blockchain technology using cancer care as an example [54].

#### Data Integration Scenario

**Figure 10:**
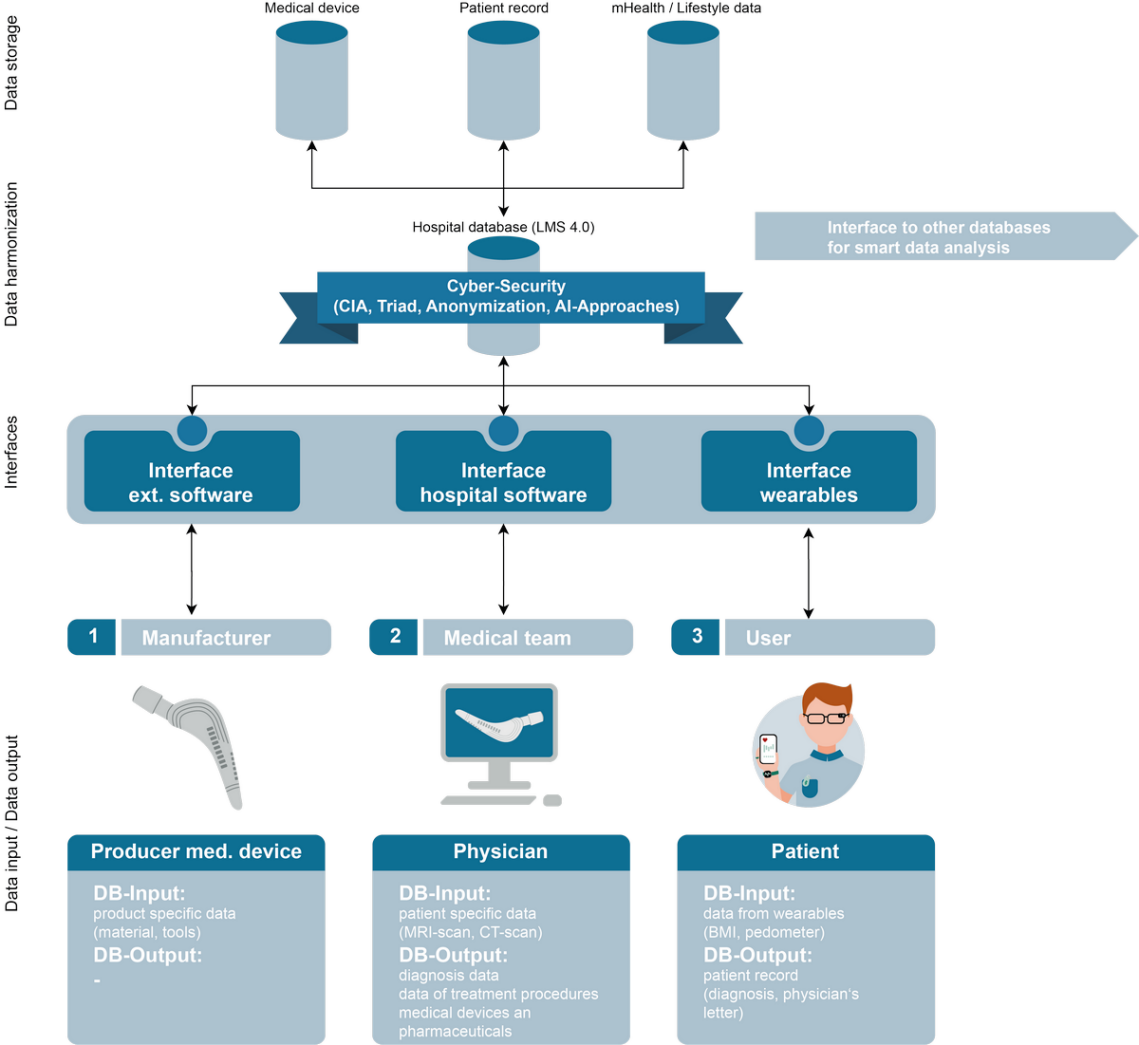
Data integration ecosystem with hierarchical data access. AI: artificial intelligence; CIA: confidentiality, integrity, availability; CT: computed tomography; DB: database; ext: external; LMS: laboratory management system; med: medical; mHealth: mobile health; MRI: magnetic resonance imaging.

Using the hospital database LMS 4.0 (Figure 8), all individual elements of the data ecosystem are presented and explained in reference to Figure 10. In fact, the data ecosystem is divided into the following 4 levels, which are distinguished for functional structuring: “data storage,” “data harmonization,” ”interfaces,“ and ”data input/data output.“ The ”data storage“ level contains different relational databases, which, again, contain medically relevant data (medical device [see the Technical Focus Data section], patient record [see the Medical Focus Data section], and health [see the Social Focus Data section]). Data preprocessing is performed at the ”data harmonization“ level (see Figures 8 and 10), which means that incoming data are adapted to the requirements of the LMS 4.0 database structure (Figure 8) and sorted, while outgoing (anonymized) data (eg, via the data integration center) are transferred via defined data exchange procedures with strictly recorded accesses. In the underlying, but closely related, ”interfaces“ level (Figure 10), interfaces are established by extensible middleware to communicate with the ”hospital database“ (see Figure 8). This enables the integration of data users from different domains and querying data from the database. The 3 levels ”interfaces,“ ”data harmonization,“ and ”data storage“ are subjected to the CIA (confidentiality, integrity, availability) triad and ensure the functionality of the system. The ”data input/data output“ level connects the ”hospital database“ with the environment. For example, manufacturers of medical devices can transfer product-specific data into the database to make the data available to hospital staff (see Figures 3 and 10). Likewise, patients can store their vital signs from wearables, for example, in this database to support long-term examinations or enable access in medical emergencies through attribute-based access control (see the Role Concept for Secure Data Access section). At the same time, patients can see their EHR, read digital doctor's notes, or view exam results. Physicians have interfaces to both connect medical exam machines to the database and write data; medical staff can also store information, and the data can be viewed hospital-wide and processed with appropriate IT systems.

## Discussion

### Principal Findings

The connection of different data lakes, beginning with implant design including the entire manufacturing to the medical treatment process as well as the tracking of lifetime characteristics inside of a data integration ecosystem, shown in this paper can disrupt today’s health care system, leading to a cost-efficient, personalized system. The strict hierarchical data access concept based on a shell-embedded role model can be used to handle highly sensitive data and as a template to help clarify legal issues.

The overriding goal is to use digitization to improve the networking of interdisciplinary domains and to create secure interfaces for exchange as a prerequisite for intelligent data analysis. For this purpose, a representative hip implant application scenario was chosen due to an existing network of social, medical, and technical domains. Moreover, the interaction resulted from a skill- and experience-based union of implant and recipient results in an individual constellation that is subject to change over time. A key element in the resulting constellation is the UDI based on an inherent feature, which can be read out noninvasively after implantation. The permanently readable feature acts as a key to technical focus data, which represent testable or documented properties that are made available by the provider and are of direct or downstream interest. Consequently, we showed how the technical focus data can be integrated into existing data ecosystems. This, however, was only approached at the hospital level, which is explained by the unclear legal framework and the missing data infrastructure for a broader context. Nevertheless, the consolidation of distributed databases in a single technology solution is a scalable concept that can be transferred from a single hospital to a global solution. Another important aspect is that the introduced hierarchical data access is based on a shell-embedded role model and staggered user rights. Here, the attribute-based access control shall be emphasized because this represents nonrigid boundary conditions in preparation for future regulations. The selected user profiles and the granted rights, on the other hand, are only examples that are up for discussion and need to be specified and challenged in further research. However, the data integration scenario distinguishes 4 levels of action layering data storage, data harmonization, interfaces, and the data input/data output layer, which harmonizes the application scenario and digital ecosystem. Nevertheless, future research must show how real benefit can be created through data linkage and how this can be monetized. Balancing the personal rights of the individual while achieving sustainable technological innovation is seen as the central challenge, which must be faced in a global context.

### Conclusions

Personalized medicine requires cross-domain linkage of data, which, in turn, requires an appropriate data infrastructure and adequate hierarchical data access solutions.

Hip implant is a prime example of the usefulness of cross-domain linkage of data because it bundles social factors of the individual patient, medical aspects in the context of the implantation, and technical aspects of the implant.

UDI in terms of inherent identifiers can be the key to (selective) long-term data access especially if the postoperative readout is guaranteed.

SLM and electron beam melting make it possible to integrate features already inherent in the design process to close the traceability gap.

It is necessary to open existing databases using suitable interfaces for secure integration of data from end devices (eg, wearables or end users) and to assure availability through suitable access models (role-based, attribute-based, hybrid) while guaranteeing long-term independent data persistence.

A suitable strategy requires the combination of technical solutions from the areas of data storage, cryptographic procedures, and software engineering as well as organizational changes among the actors involved (eg, hospital staff, implant manufacturers, patients).

Holistic approaches require interdisciplinary cooperation and cross-domain data spaces, while innovative approaches and services must be developed prior or parallel to the ongoing clarification of the legal framework conditions.

To provide viable and transferable solutions at the time of legal clarification, cross-domain lighthouse projects are needed to assure the timely availability of digital business models, suitable data alliances, and an adequate digital infrastructure.

## Abbreviations

AM: additive manufacturing
API: application programming interface
CIA: confidentiality, integrity, availability
CT: computed tomography
EC: eddy current
ETL: extract, transform, load
HIS: hospital information system
IT: information technology
LMS: Laboratory Management System
MDR: Medical Device Regulation
mHealth: mobile health
PHR: personal health record
SLM: selective laser melting
UDI: unique device identification
US: ultrasound
